# Ammonium Toxicity and Potassium Limitation in Yeast 

**DOI:** 10.1371/journal.pbio.0040351

**Published:** 2006-10-17

**Authors:** David C Hess, Wenyun Lu, Joshua D Rabinowitz, David Botstein

**Affiliations:** 1 Lewis-Sigler Institute for Integrative Genomics, Princeton University, Princeton, New Jersey, United States of America; 2 Department of Molecular Biology, Princeton University, Princeton, New Jersey, United States of America; 3 Department of Chemistry, Princeton University, Princeton, New Jersey, United States of America; Duke University Medical Center, United States of America

## Abstract

DNA microarray analysis of gene expression in steady-state chemostat cultures limited for potassium revealed a surprising connection between potassium and ammonium: potassium limits growth only when ammonium is the nitrogen source. Under potassium limitation, ammonium appears to be toxic for Saccharomyces cerevisiae. This ammonium toxicity, which appears to occur by leakage of ammonium through potassium channels, is recapitulated under high-potassium conditions by over-expression of ammonium transporters. Although ammonium toxicity is well established in metazoans, it has never been reported for yeast. To characterize the response to ammonium toxicity, we examined the filtrates of these cultures for compounds whose excretion might serve to detoxify the ammonium (such as urea in mammals). Using liquid chromatography–tandem mass spectrometry to assay for a wide array of metabolites, we detected excreted amino acids. The amounts of amino acids excreted increased in relation to the severity of growth impairment by ammonium, suggesting that amino acid excretion is used by yeast for ammonium detoxification.

## Abstract 2

An amino acid excretion mechanism is identified in yeast; this stress response helps purge excess nitrogen when ammonium ion toxicity is encountered (presumed to leak through potassium channels) in potassium-limiting conditions.

## Introduction

Maintaining intracellular metabolites within acceptable levels in the face of an ever-changing environment is one of the great challenges faced by microorganisms. This maintenance of metabolic homeostasis is achieved largely by controlling the activities and concentrations of enzymes and transporters. One important means of controlling enzyme concentrations is transcriptional regulation [1–3]. In the wake of the genomic sequence, comprehensive measures of transcript levels can be readily obtained. By combining these data with other high throughput measurements, such as metabolite profiling [4], one can hope to capture how various cellular systems are interacting to maintain metabolic homeostasis (cf., reference [5]).

Although there is a relatively rich literature describing microbial growth regulation in response to changes in limiting nutrients, responses to limiting ion concentrations have not been extensively studied. In this paper we show how genomic analysis led to the realization that potassium limitation is intimately intertwined with ammonium toxicity. Specifically, we demonstrate how the combined power of microarray analysis of transcripts and mass spectrometry–based analysis of small molecule metabolites led unexpectedly from experiments on potassium limitation to three major findings regarding ammonium: (1) growth of Saccharomyces cerevisiae is limited at low concentrations of potassium ions by ammonium toxicity; (2) ammonium toxicity also occurs, even in the presence of high concentrations of potassium ions, if ammonia transporters are constitutively expressed; and (3) the physiological response of yeast to ammonium toxicity, however achieved, involves excretion of large quantities of amino acids.

Potassium ions (K^+^; hereafter referred to as “potassium”) are the most abundant intracellular cations in most types of cells, from S. cerevisiae to humans. Intracellular levels are maintained in yeast against a steep chemical gradient, often exceeding 100-fold, with the intracellular concentration of 150 mM [6,7]. This gradient is maintained in media containing less than 1 mM potassium and has been reported to play a crucial role in a variety of cellular processes, including regulation of cell volume, intracellular pH, and protein synthesis, and prevention of the deleterious effects of sodium ions [6,8]. Understanding of the role of potassium in yeast is relatively undeveloped compared to that in mammalian systems (e.g., in neurons and cardiomyoctes).

With an initial aim of better understanding the physiological role of potassium ions in yeast, we studied the gene expression pattern of cells grown to steady state in chemostats with potassium as the limiting nutrient [9,10]. Analysis of DNA microarray data from chemostat cultures growing in apparently limiting concentrations of potassium showed a surprisingly small set of substantial gene expression changes. Yet more surprisingly, the majority of these changes involved genes implicated in nitrogen metabolism. All the known ammonium assimilation pathways of yeast were strongly down-regulated in low-potassium media. Ammonium (NH_4_^+^) was the sole source of nitrogen in these experiments; it is the preferred nitrogen source for bacteria and fungi [11]. Subsequent experiments revealed that its presence was responsible for the poor growth of the “potassium-limited” cells.

Excess ammonium is well known to cause toxicity in plant and animal systems [11]. Ammonium toxicity has been dismissed as a problem for microorganisms [12] on the rationale that regulation of ammonium transporters can prevent excess ammonium from entering the cell. The similar ionic properties (charge, radius, and hydration energies) of potassium and ammonium raises the possibility that, under “limiting potassium,” toxic amounts of ammonium might enter yeast via potassium channels as has been observed for potassium channels in other systems [13,14]. Furthermore, experiments in yeast have implicated a link between ammonium and potassium [15,16]. Below we report a series of studies confirming this suspicion. We also find via metabolite profiling that, when experiencing ammonium toxicity, yeast respond by excreting amino acids. Such amino acid excretion may constitute a rudimentary ammonia detoxification mechanism in the yeast.

## Results

**Figure 1:**
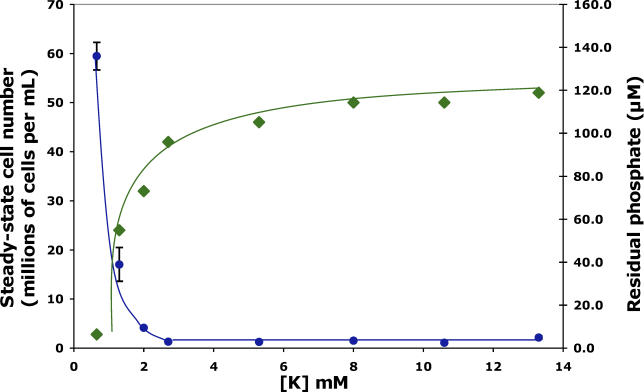
Potassium Limitation in the Chemostat Steady-state cell number (green diamonds, left axis) was measured over a potassium concentration of 0.65 mM to 13 mM. Additionally, residual phosphate at steady state (blue circles, right axis) was measured from filtrates of these chemostats.

A chance observation in phosphate-limited chemostat cultures showed that it was possible to limit growth in such cultures even further by limiting the concentration of potassium ions in the medium. To study this possibility more systematically, a series of such nominally phosphate-limited chemostats were established with decreasing amounts of potassium in the medium. These experiments were done in an otherwise typical minimal yeast medium, which happens to contain 76 mM ammonium as the nitrogen source. The results of measurements of steady-state cell number and residual levels of phosphate are shown in Figure 1. As the supplied potassium concentration fell below about 3 mM, the cell numbers (and thus the biomass) were sharply reduced, and the residual phosphate was sharply higher, indicating that phosphate is no longer limiting growth.

### Transcriptional Response to Low Potassium Implicates Ammonium

**Figure 2:**
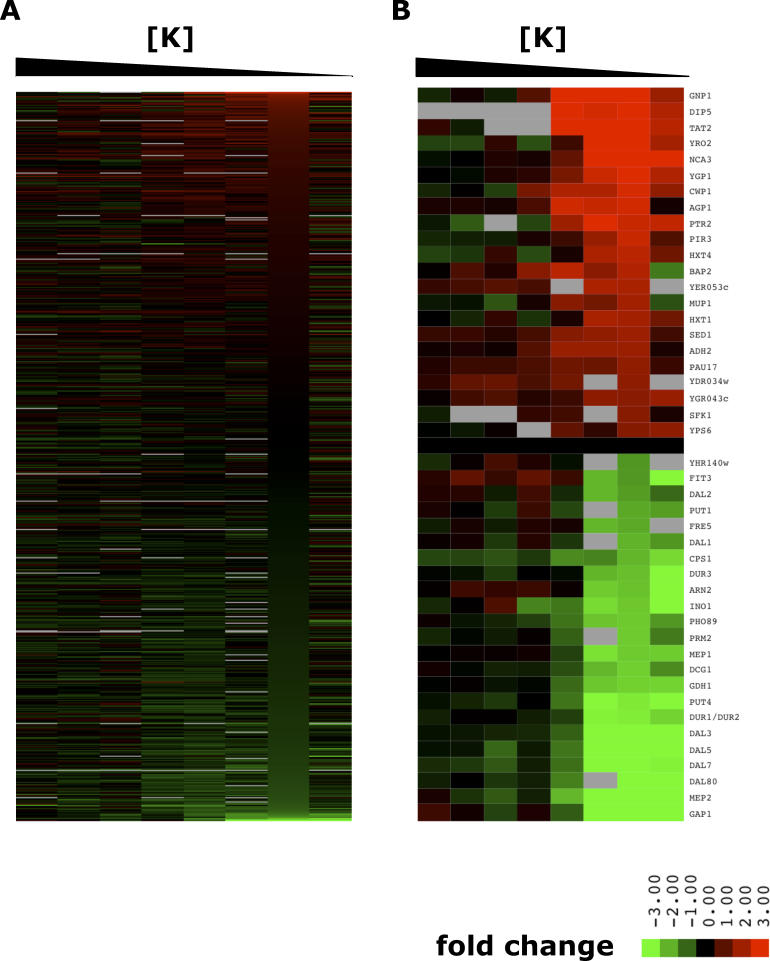
Expression Arrays Reveal Changes in Nitrogen Transporters (A) Ranked list of expression data from steady-state chemostats with different potassium concentrations (the same chemostats shown in Figure 1). The arrays use 13 mM potassium (far left) as a reference and are ranked from most induced in 1.3 mM potassium to most repressed in 1.3 mM potassium. Each row represents a single gene and each column is a single array. Red values indicate a higher expression compared to the reference, whereas green values indicate a lower expression. The intensity of the color represents the strength of the fold change and corresponds to the legend in the bottom right. (B) Genes that were 3-fold induced (top) or 3-fold repressed (bottom) in 1.3 mM potassium are shown with their gene name.

To investigate the physiological reason(s) for this apparent growth limitation by potassium, gene expression studies were carried out on the cells recovered from these chemostat cultures. The results of these gene expression studies (Figure 2; Tables 1 and 2) were remarkably informative. A surprisingly limited number of genes were strongly affected by the potassium limitation, and the great majority of these were genes known to be involved in nitrogen metabolism. The genes encoding major ammonium ion and amino acid transporters *(GAP1* and *MEP2)* were down-regulated 30-fold; virtually all other nitrogen catabolite–repressed genes were strongly down-regulated as well. The SPS-regulated amino acid permeases were all strongly up-regulated. Notably there was a more than 10-fold up-regulation of high-affinity and high-capacity permeases for glutamine *(GNP1),* for glutamate and aspartate *(DIP5),* and for tryptophan and tyrosine *(TAT2)*. Genes encoding the other amino acid and oligopeptide permeases in this group *(AGP1, PTR2, BAP2,* and *MUP1)* were up-regulated 5-fold or more. Together these permeases facilitate transport of all the amino acids except arginine.

**Table 1:**
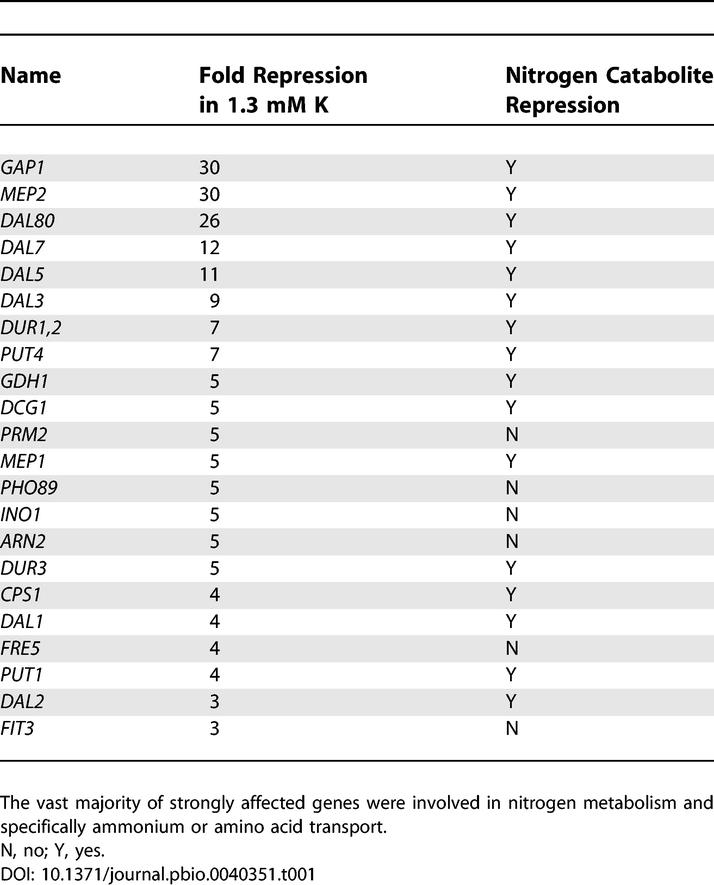
Genes That Were 3-Fold Repressed Are Shown with Their Gene Name, Fold Change in 1.3 mM Potassium, and Functional Information

**Table 2:**
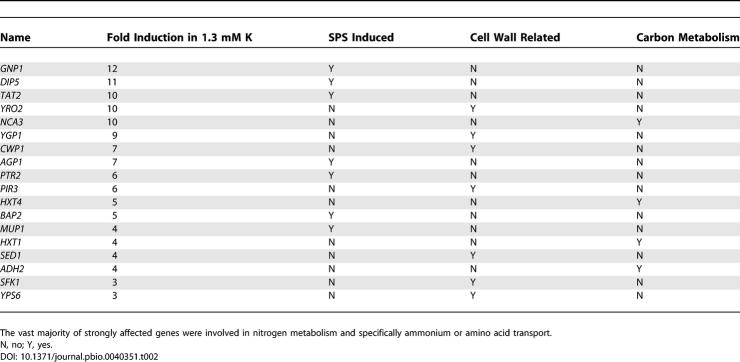
Genes That Were Induced 3-Fold Are Shown with Their Gene Name, Fold Change in 1.3 mM Potassium, and Functional Information

**Figure 3:**
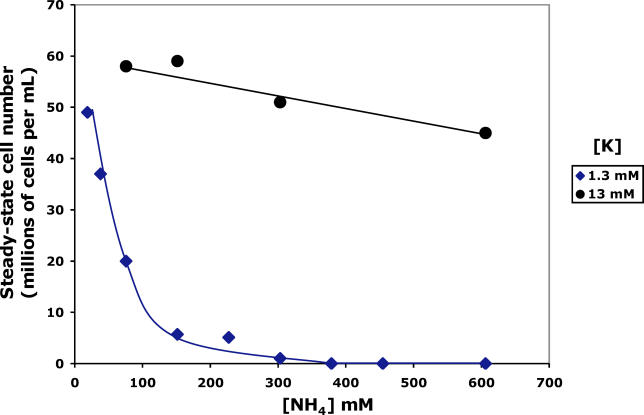
Ammonium Is Toxic to Yeast in Low Potassium Steady-state cell number for 1.3 mM potassium (blue diamonds) or 13 mM potassium (black circles) medium was measured over an ammonium concentration of 19 mM to 606 mM.

These results prompted another series of chemostat cultures, this time with two different fixed levels of supplied potassium (either “low” [1.3 mM] or “normal” [13 mM]) and varying levels of ammonium. The term “low” is used for 1.3 mM potassium, because typical yeast media contain 13 mM potassium. However, 13 mM potassium is higher than both seawater (0.5 mM potassium) and human serum (4 mM potassium), suggesting that in reality, 1.3 mM potassium may be more reflective of typical yeast environmental conditions than 13 mM potassium [17,18]. The results (Figure 3) clearly showed a decline of cell numbers (and hence biomass) that was a strong function of the ammonium concentration in 1.3 mM potassium media, but not 13 mM potassium media. Indeed, in 1.3 mM potassium media at levels greater than 300 mM ammonium, the yeast were entirely unable to maintain steady-state chemostat growth at reasonable dilution rates. These results led to the hypothesis that toxic levels of ammonium were leaking into the cell via potassium channels.

**Figure 4:**
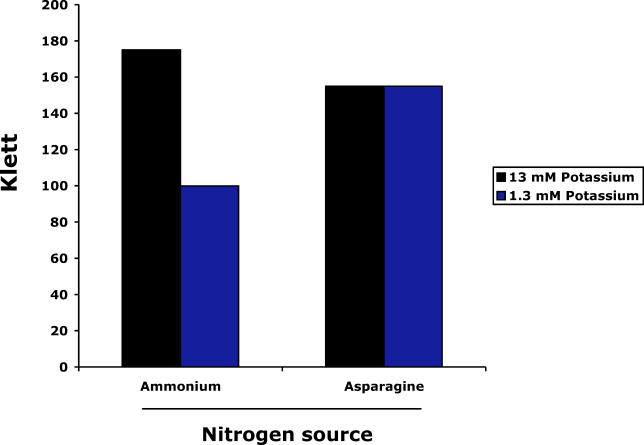
Toxicity Is Specific to Ammonium as a Nitrogen Source Chemostats were run in high (13 mM, black bars) or low (1.3 mM, blue bars) potassium with either ammonium (left) or asparagine (right) as the sole nitrogen source. As demonstrated before, steady-state cell mass (measured here by Klett Colorimeter) decreased with lower potassium when ammonium was the nitrogen source. However, if asparagine was used as the nitrogen source, then reduced potassium had no effect on steady-state cell mass.

The above results suggested that the reduction of biomass in low-potassium chemostats would be specific to ammonium as a nitrogen source. To test this, we compared a set of “normal” (13 mM) and “low” (1.3 mM) potassium chemostats with asparagine versus ammonium as the nitrogen source (Figure 4). Only the ammonium-fed cells were sensitive to low potassium, bolstering the hypothesis that the biomass reduction in low-potassium chemostats was due to ammonium toxicity.

### Excess Ammonium Results in Amino Acid Excretion

**Figure 5:**
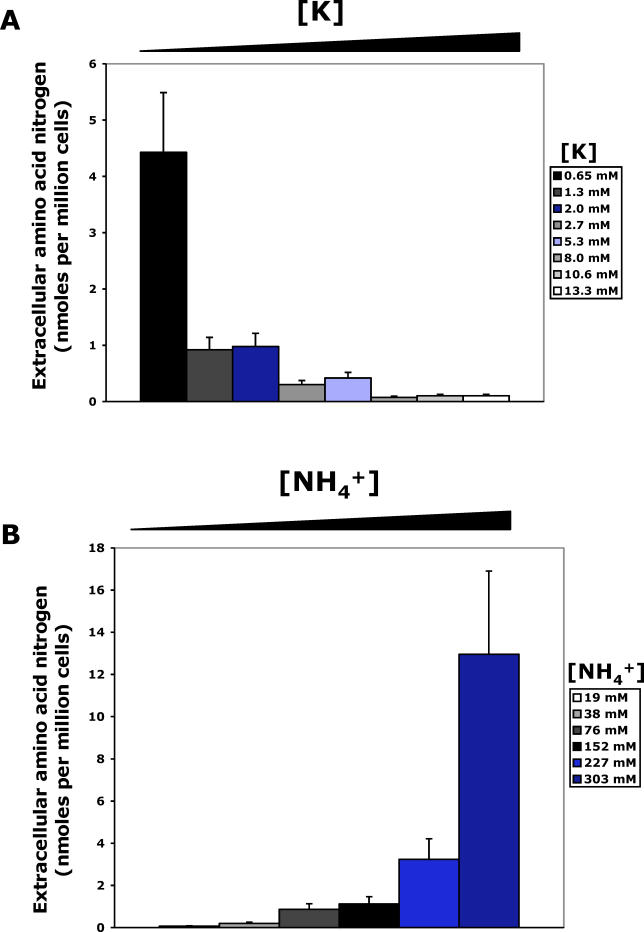
Ammonium Toxicity Induces Amino Acid Excretion LC-MS/MS was used to analyze filtrates from steady-state chemostats. The detected amino acids were expressed in nmoles based on standards (Materials and Methods). The detected amino acids were scaled for number of excreted nitrogen molecules per amino acid (i.e., one for alanine and two for glutamine) and expressed in nmoles excreted nitrogen per million cells. (A) Excreted amino acids for a range of potassium (same as the chemostats shown in Figure 1). (B) Excreted amino acids were measured for a range of ammonium in 1.3 mM potassium (same as the chemostats shown in Figure 3).

Most metazoans excrete one or more nitrogen-containing organic compounds to maintain nitrogen homeostasis [19]. We hypothesized that S. cerevisiae could be excreting nitrogen-containing organic compounds in an effort to detoxify ammonium. Accordingly, we used liquid chromatography–tandem mass spectrometry (LC-MS/MS) to test the filtrates of steady-state chemostats for the presence of dozens of different, known N-containing metabolites. This approach revealed significant amounts of amino acids in the filtrates of cells suffering ammonium toxicity (Figure 5).

The amount of excreted amino acids per cell rose as ammonium toxicity became more severe. We observed increased excretion both when we reduced potassium with a fixed concentration of ammonium (Figure 5A) and when we increased ammonium at a fixed concentration of potassium (Figure 5B). Although we were able to detect significant signal for 14 of the 20 amino acids, the majority of extracellular organic nitrogen was contained in five amino acids: alanine, valine, proline, glutamate, and glutamine (Table 3). From these data we hypothesize that excretion of amino acids can be used to detoxify excess ammonium inside S. cerevisiae.

**Table 3:**
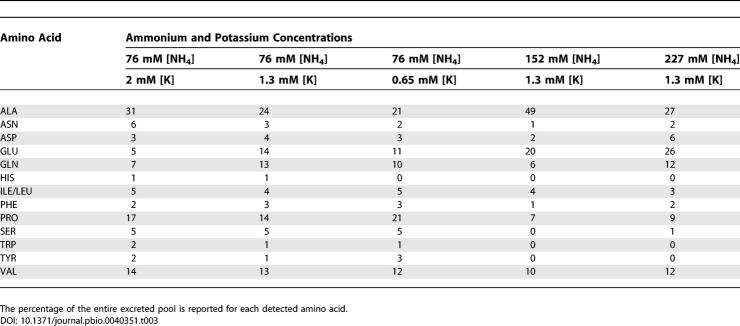
Profile of Excreted Amino Acids

Besides the physiologically reasonable relationship between the growth impairment by ammonium toxicity and the level of amino acids found in the filtrates, there are three reasons for believing that the presence of amino acids in these filtrates is due to bona fide excretion. First, our chemostat medium was a completely defined minimal medium containing no amino acids, so the amino acids must have been synthesized by the yeast. Second, we tested cell viability at concentrations of potassium from 13.3 mM to 0.65 mM and found cell viability above 85% in all cases, making it unlikely that cell lysis was the source of the significant levels of amino acids in the filtrate. Third, using the isotope spiking system described in the Materials and Methods, we could estimate the total amounts of three different amino acids inside the cells (i.e., in the extract) relative to the medium (i.e., in the filtrate) in a 1.3 mM potassium chemostat culture. From these numbers (1:10 extract:filtrate for glutamine; 1:4 for alanine; and 1:3 for glutamate) it is clear that at least three fourths of the cells would have had to lyse to account for the amino acids in the filtrate, a finding completely inconsistent with the >90% viability observed in that 1.3 mM potassium chemostat (Figure S1).

**Figure 6:**
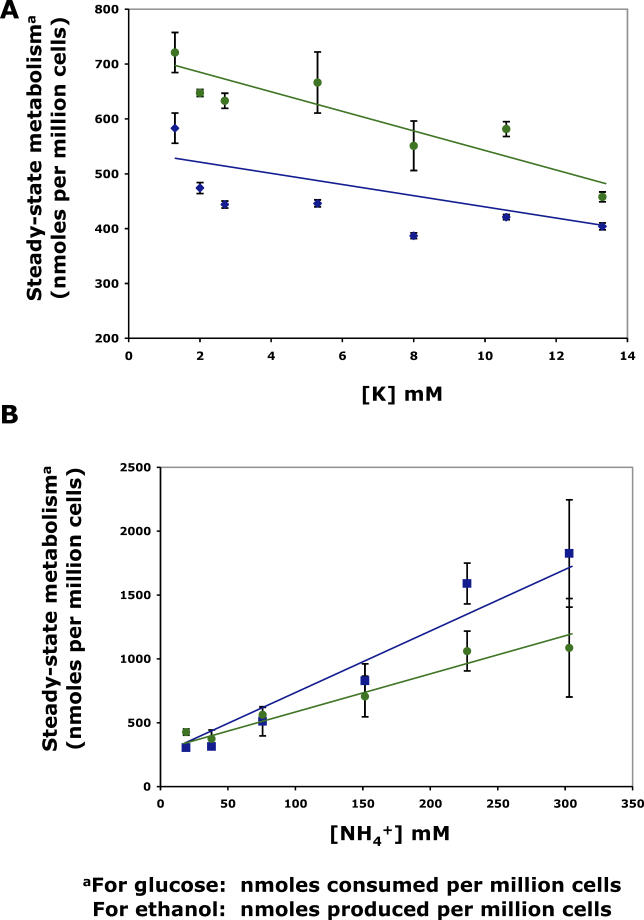
Ammonium Toxicity Is Correlated with Increased Glucose Utilization and Ethanol Production Specific glucose utilization and ethanol production were measured from steady-state chemostat cultures. Specific glucose utilization (blue points) is expressed in nmoles consumed per million cells, and specific ethanol production (green points) is expressed in nmoles produced per million cells. (A) Glucose utilization and ethanol production from steady-state chemostats with different concentrations of potassium (same as the chemostats shown in Figure 1). (B) Glucose utilization and ethanol production were measured from steady-state chemostats with different concentrations of ammonium at 1.3 mM potassium (same as the chemostats shown in Figure 3).

Nitrogen assimilation into amino acids is expensive both energetically and in terms of amino acid carbon skeletons. Consistent with this, glucose consumption increased modestly under low-potassium conditions at 76 mM ammonium (Figure 6A), and dramatically as ammonium concentration was increased yet further at 1.3 mM potassium (Figure 6B). Ethanol production increased in parallel with glucose consumption, consistent with idea that these cells were fermenting more glucose for energy.

### Ammonium Toxicity in Low Potassium Is Not Dependent on Strain Background

**Figure 7:**
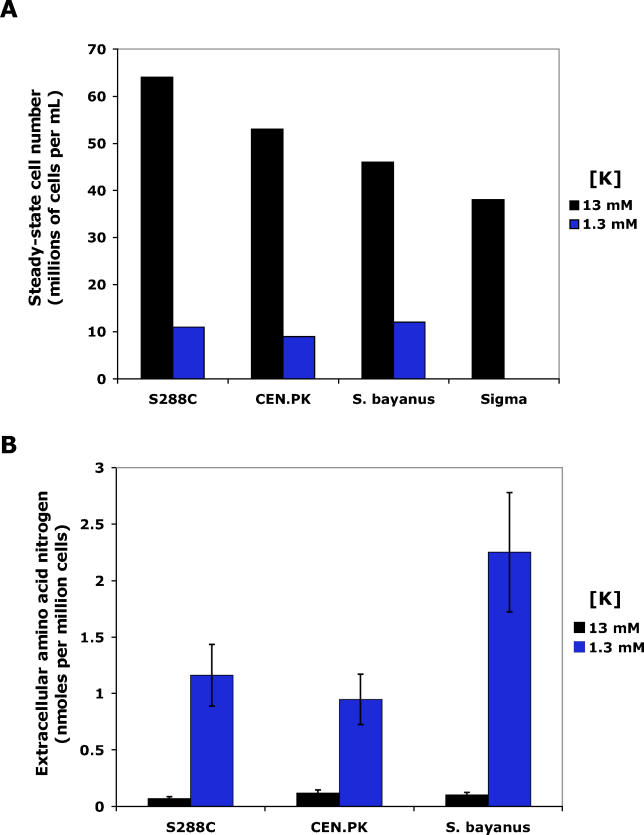
Ammonium Toxicity and Excretion of Amino Acids Is Observed across a Variety of *Saccharomyces* Strains (A) Chemostats were run in high (13 mM, black bars) or low (1.3 mM, blue bars) potassium with ammonium as a nitrogen source for the indicated *Saccharomyces* strains. As observed in Figure 1 and Figure 4 for S228C, all strains tested showed decreased steady-state biomass in low potassium. The Sigma strain washed out of the chemostat in 1.3 mM potassium, indicating that it could not maintain a growth rate equal to the dilution rate. (B) LC-MS/MS was used to analyze filtrates as described in Figure 5. Filtrates from steady-state chemostats from (A) were analyzed, and increased amino acid excretion was observed for all low-potassium chemostats. The Sigma strain was not analyzed because it washed out in low potassium.

To ensure that our observations were not dependent on the S288C background, a series of high (13 mM) and low (1.3 mM) potassium chemostats were brought to steady state for three other strains of yeast. Saccharomyces bayanus (considered a separate species that diverged ca. 20 million y ago [20]) and two additional laboratory strains of S. cerevisiae (CEN.PK and Σ) were tested (Figure 7). All strains had significantly decreased steady-state biomass in low potassium; strain Σ was unable to achieve steady state and washed out of the chemostat (Figure 7A). For strains able to maintain steady state in both high and low potassium, filtrates were examined for the presence of amino acids (Figure 7B). All strains had increased excretion of amino acids in low potassium; it is notable that *S. bayanus,* the most nearly “wild” strain, showed this phenotype most strongly. These data suggest that the response to ammonium toxicity is a robust metabolic program conserved among several variants of *Saccharomyces* known to have significant phenotypic differences related to nitrogen metabolism [21].

### Ammonium Toxicity and Amino Acid Excretion Caused by Over-Expression of Ammonium Transporters

To test our hypothesis that ammonium toxicity was occurring via an unregulated leakage of ammonium ions, we undertook to provide the cells with unregulated ammonium ion influx independent of potassium channels. To this end, we constructed strains in which we replaced the promoters of known ammonium transporters with a strong, galactose-inducible promoter. S. cerevisiae has three genes encoding ammonium transporters *(MEP1, MEP2,* and *MEP3),* each of which is normally under tight transcriptional regulation in response to nitrogen levels. *MEP1* and *MEP3* encode high-flux, low-affinity transporters, whereas *MEP2* encodes the low-flux, high-affinity transporter [22]. We produced strains that contained each of these genes individually under the control of a galactose promoter. In addition we placed the galactose promoter construct in front of a dubious open reading frame (ORF), *YIL028w,* as a control strain. The construction of these strains allowed us to examine how S. cerevisiae would respond to a strong ammonium influx that could not be regulated by ammonium or other internal nitrogen metabolite levels.

**Figure 8:**
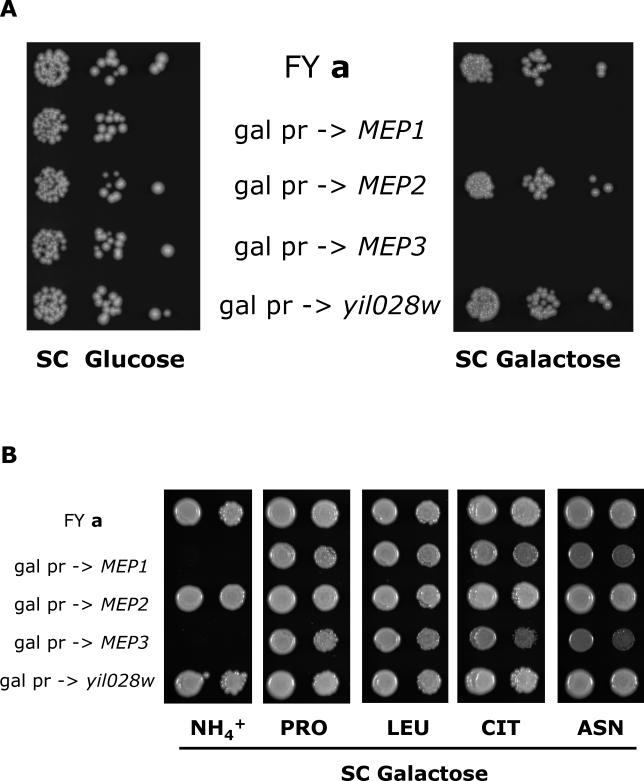
Over-Expression of High-Flux Ammonium Channels Causes Lethality on Ammonium Growth was assessed by frogging a dilution series (from 10^7^ cells/ml to 10^3^ cells/ml) onto SC plates with either 2% glucose or 2% galactose as the carbon source. Nitrogen sources were as follows: 5-g/l (NH_4_)_2_SO_4_ (NH_4_), 5-g/l asparagine (ASN), 2.5-g/l citrulline (CIT), 10-g/l proline (PRO), or 10-g/l leucine (LEU). The most informative dilutions are displayed for each medium (10^7^–10^5^ cells/ml for [A] and 10^5^–10^4^ cells/ml for [B]). Plates were incubated for 3 d at 30 °C. (A) Strains frogged on plates with ammonium as a nitrogen source with either glucose (left) or galactose (right) as the carbon source. (B) Strains frogged on plates with galactose as the carbon source and a series of nitrogen sources. gal pr, galactose promoter.

We found that expression from the galactose promoter of the high-flux ammonium transporters *(MEP1* and *MEP3),* but not the low-flux one *(MEP2),* caused lethality (Figure 8A) in ammonium-containing minimal media. To test whether this lethality was due to ammonium toxicity, we induced the promoters with galactose in the presence of several nitrogen sources (Figure 8B). When asparagine, leucine, proline, or citrulline were used as a nitrogen source, strains expressing the *MEP1* and *MEP3* from the galactose promoter grew well, and showed no lethality, in the presence of galactose. These data demonstrate that an unregulated influx of ammonium per se is lethal to S. cerevisiae.

**Figure 9:**
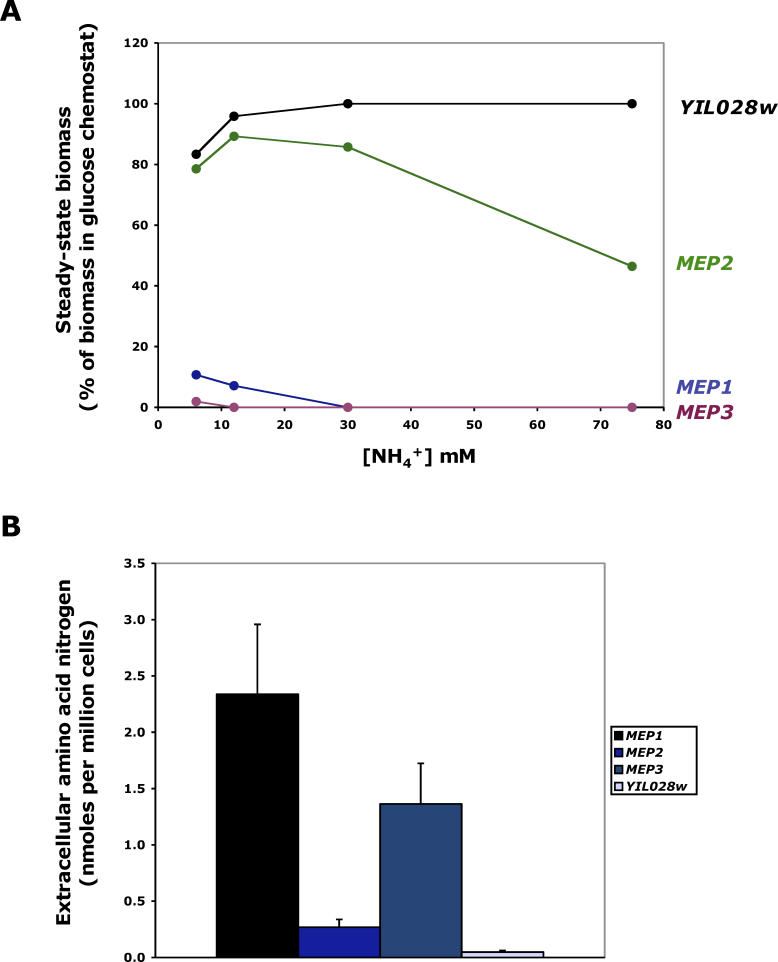
Amino Acid Excretion Is Induced at Steady State when Ammonium Channels Are Over-Expressed (A) Each of the *MEP* over-expressing strains was tested in the chemostat at high (13 mM) potassium under a range of ammonium concentrations (6 mM to 76 mM) with galactose as the carbon source. Steady-state biomass is reported as percentage of the biomass in standard phosphate-limited medium with glucose as the carbon source. *MEP1* (blue) and *MEP3* (pink) only maintained steady state at the lowest concentrations of ammonium with galactose as the carbon source. (B) Amino acid excretion was measured as before (Figure 5) in 6 mM ammonium medium with galactose as the carbon source.

To examine further the *MEP* over-expressing strains, we grew them in a phosphate-limited chemostat with galactose as a carbon source (Figure 9A). The media contained high (13 mM) potassium and standard (76 mM) ammonium concentrations. Nevertheless, *MEP1* and *MEP3* over-expressing strains were not able to achieve steady state. Although *MEP2* could reach steady state, the biomass was significantly less than the control strain for ammonium concentration in excess of 70 mM, revealing a weaker *MEP2*-induced ammonium toxicity phenotype not captured on plates. To achieve steady-state chemostats for *MEP1* and *MEP3,* ammonium concentration in the medium had to be reduced to levels at which nitrogen and not phosphate was limiting for *MEP2* and the control strain. This sensitivity to ammonium demonstrates that we can recreate the growth defects associated with low potassium by over-expressing ammonium transporters.

We examined the filtrates by LC-MS/MS from the *MEP* over-expressing strains in 6 mM ammonium (Figure 9B). This analysis revealed that the *MEP1* and *MEP3* strains were excreting amino acids at a much higher rate than the control *(YIL028w)* strain. These data indicate that our *MEP1* and *MEP3* over-expressers not only recapitulate the ammonium sensitivity of strains grown in low potassium, but also respond to this ammonium toxicity by excreting amino acids. Additionally, the *MEP2* over-expressing strain was excreting significantly more amino acids than the control strain even though the *MEP2* strain had a similar steady-state biomass to the control at the low ammonium concentration used in this experiment. These *MEP2* data suggest that under certain conditions, amino acid excretion is able to buffer excess nitrogen to retain wild-type growth.

## Discussion

The central biological finding of this study is that there are circumstances under which S. cerevisiae is subject to ammonium toxicity. We found this toxicity under two circumstances: lower than standard concentrations of potassium in the medium, and over-expression of genes *(MEP1* and *MEP3)* encoding ammonium ion transporters.

We believe that ammonium toxicity has gone undiscovered in yeast and bacterial systems largely because standard defined growth media contain very high (likely supra-physiological) potassium concentrations. For example, although seawater contains only 0.5 mM potassium [17] and human serum only 4 mM [18], the medium in which C. glutamicum was recently tested for ammonium toxicity contained 92 mM potassium [12]. Thus it may well be that ammonium toxicity in a variety of bacterial and fungal systems has been masked by the routine use of these very high potassium concentrations.

 Although ammonium toxicity in low-potassium medium greatly affected the steady-state biomass in the chemostat, gene expression studies did not indicate a strong stress response [5,23]. Instead, a specific and coordinated transcriptional program that mainly affected nitrogen metabolism (specifically ammonium and amino acid transport), and a few cell wall–related functions. We interpret the transcription response as part of *Saccharomyces*' natural homeostatic repertoire.

**Figure 10:**
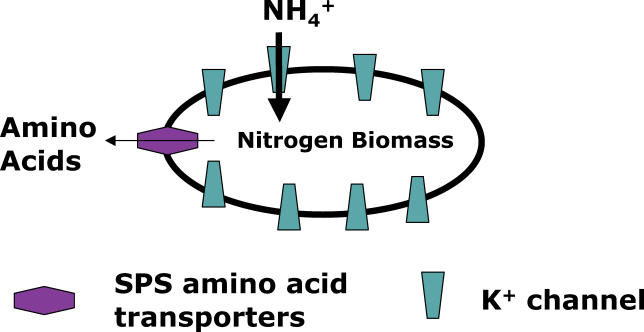
Model for Ammonium Toxicity and Detoxification in Yeast We believe the results of this paper support the following model. If ammonium is present in high concentrations in the environment, then ammonium ions can enter the cell unregulated via potassium channels. Although most of the ammonium can be taken up into new biomass (if excess carbon and other nutrients are available), the unregulated flux creates an excess of internal ammonium that becomes toxic. To reduce internal ammonium levels, amino acids are excreted (most likely through the SPS amino acid transporters). The nitrogen affixed to amino acids will not be taken up through the potassium channels and is thus detoxified with respect to the cell.

We believe all our observations are consistent with the following physiological interpretation (Figure 10).

First, ammonium ions and potassium ions are both transported by potassium transporters; under low (but physiologically reasonable) potassium concentrations; this results in a toxic inflow of ammonium ions when the latter are present in large concentrations in the medium. At the relatively very high concentrations of potassium found in standard laboratory media, this “leak current” of ammonium ions is small and tolerable.

Second, the SPS amino acid transporters are somehow induced at high internal ammonium levels, facilitating the excretion of amino acids, which appears to be the major (possibly the only) means of ammonium detoxification available to *Saccharomyces*. Besides the transcriptional evidence for this role, it is notable that, of the 15 amino acids that we can readily detect from extracts of yeast cells, the only one that was found not be excreted was arginine, the sole amino acid that is not a substrate for the SPS system.

### Integrating Systems Biology Techniques to Discover New Physiology

The discovery of ammonium toxicity in S. cerevisiae provides an example of the utility of systems biology techniques for making unexpected and yet fundamental biological discoveries. In this case, microarray analysis aimed at investigating potassium limitation surprisingly revealed that all the known ammonium assimilation pathways of yeast were strongly down-regulated. Investigation of this puzzling result led us to discover ammonium toxicity, a basic component of yeast biology that had been missed despite decades of study of related nutrient perturbations. Furthermore, the discovery of excreted amino acids would have been unlikely without metabolic profiling of the filtrate.

Unlike animals, which excrete urea or uric acid in response to nitrogen toxicity, yeast appear to use the simpler expedient of excreting amino acids to reduce the intracellular ammonium concentration. Testing directly for urea or uric acid would have yielded a negative result, and trying to assay individually for all the nitrogen-containing organic compounds in yeast would have been impractical. Thus, although genomic tools to date have been used most commonly to bring global understanding to well-studied biological processes (e.g., to determine the overall extent of transcriptional changes involved in a particular biological response), the present study highlights the value of these tools for making focused biological discoveries more efficiently.

### Yeast and Bacteria May Show Similar Links between Limiting Potassium and Ammonium Toxicity

Although ammonium toxicity has to our knowledge never been previously reported in microbes, evidence describing a link between potassium and ammonium has been noted previously. Rodriguez-Navarro and Ramos [16] found that the minimal potassium requirement for optimal growth of yeast was 100-fold lower if ammonium was not used as a nitrogen source. As indicated above, our results and interpretation readily account for this observation.

Buurman et al. [24] found evidence that ammonium could replace potassium in certain bacteria, i.e., that growth on limiting potassium improved with up to 100 mM ammonium as the nitrogen source. This result is opposite to those found here for yeast. However, there were certain similarities between the studies of Buurman et al. [24] and our own, including increased glucose utilization and glycolytic waste-product excretion (acetate or ethanol, depending on the organism) as increasing ammonium is added to potassium-limited chemostats. Also similar to our studies in yeast, Buurman et al. [25] found evidence for ammonium entry into bacteria via potassium channels. Nevertheless, future work is required to explore whether our key findings of ammonium toxicity and ammonium detoxification via amino acid secretion apply also to any prokaryotes.

### Ammonium Toxicity Is a New Component in the Complex Regulatory Network for Nitrogen Metabolism

The metabolic and regulatory processes involved in sensing and utilizing nitrogen comprise a complex biological network (for review: [26]). Ammonium plays a central role in this network. High levels of ammonium activate nitrogen catabolite repression which represses nitrogen uptake from the environment [27]. The high-affinity ammonium transporter (the product of the *MEP2* gene) is required for pseudohyphal differentiation upon nitrogen starvation [28]. Furthermore, ammonia excretion has been suggested as a signaling mechanism in long-term colony growth [29].

The response to ammonium toxicity we describe here in high-ammonium and low-potassium conditions employs known features of the nitrogen metabolic and regulatory network of yeast. As we have shown, nitrogen catabolite repression is strongly activated. In contrast, expression of the SPS-induced amino acid transporters is strongly up-regulated. The literature offers two kinds of explanations for this phenomenon: first, it is well established that amino acids in the medium induce the SPS system [30,31], and we have shown above that amino acids are indeed excreted; second, it has been reported that the SPS system participates in the production of ammonia pulses implicated in colony signaling [32]. Currently, we favor the relatively simple idea that excreted amino acids induce the SPS system, producing a positive-feedback loop because the induction facilitates further excretion; however, direct participation of an ammonia signaling system cannot be ruled out. In this connection, it is worth noting that tiny concentrations of amino acids consistent with leakage (e.g., 1 μM leucine) suffice to induce the SPS system [30,31].

### Role of the SPS-Induced Amino Acid Transporters

The spectrum of amino acids that are excreted in response to ammonium toxicity is consistent with our hypothesis of the central role of the SPS transporters: out of the 15 amino acids that we routinely detect from yeast extracts, only arginine is not detectably excreted. The *CAN1* gene encodes the only transporter of arginine, and *CAN1* is not among the broad range of transporter genes induced by the SPS pathway [33,34]. The conspicuous absence of arginine from the filtrate thus supports the idea that the SPS system is the primary means by which amino acids leave the cells.

Although the SPS system has traditionally been considered a means of amino acid import, the transporters are passive and thus must, based on the second law of thermodynamics, be bidirectional. Consistent with this, we have recently obtained additional evidence supporting the ability of the SPS system to export amino acids (D. C. Hess, unpublished results): use of aspartate, a known inducer of the SPS system, as a nitrogen source leads to leakage of a broad spectrum of amino acids into the media, whereas use of proline, which does not induce the SPS system, does not [30,31,35–38].

Other potential routes to activation of the SPS system are suggested by the previous findings that amino acid excretion in yeast can occur in conditions of deregulated anabolism or impaired catabolism [22,39], and that the transporter encoded by the *AQR1* gene can excrete amino acids [40]. Although Aqr1p might play a role in initiating amino acid excretion, we think it is unlikely to be the major contributor, as its transcription does not change significantly under conditions of ammonium toxicity, and the excreted amino acid profile of nitrogen toxicity differs from the excreted amino acid profile of an *AQR1* over-expressing strain [40].

Beyond excreting amino acids, *Saccharomyces* might be able to detoxify excess nitrogen by excreting ammonium directly. Excretion of ammonium by the transporters encoded by the *ATO1, ATO2,* and *ATO3* genes has been reported [41]. Interestingly, transcription of one of these transporters *(ATO3)* is regulated by the SPS signaling cascade [42]. We observe a modest 2-fold induction of *ATO3* under low-potassium conditions.

### Conclusion

The experiments described above show that, at physiological concentrations of potassium, excess ammonium is toxic to three strains of S. cerevisiae and to S. bayanus as well. Our data show that detoxification involves massive excretion of amino acids, more or less in proportion to the degree of ammonium toxicity. Based on its induction, and the spectrum of amino acids excreted, we believe that the bulk of the amino acid excretion occurs via the SPS-induced transporters. Although we believe that, in the natural environment, excess ammonium leaks into the cells through the potassium channels, both the toxicity of ammonium and the amino acid excretion response can be recapitulated by artificially inducing excess ammonium inflow with *MEP* transporter over-expression. Taken together these data uncover a new facet of nitrogen metabolism in yeast and demonstrate that, like higher eukaryotes, yeast detoxify ammonium by excretion of nitrogen-rich compounds.

## Materials and Methods

### Yeast strains and genetic methods.

All S. cerevisiae strains used in this study (Table 4) are derived from a prototrophic GAL2^+^ derivative of S288C [43]. Standard methods for mating, sporulation, transformation, and tetrad analysis were used [44]. We constructed derivatives of the *MEP* genes that replaced the native promoter with a galactose-inducible promoter with constructs previously described [45].

**Table 4:**
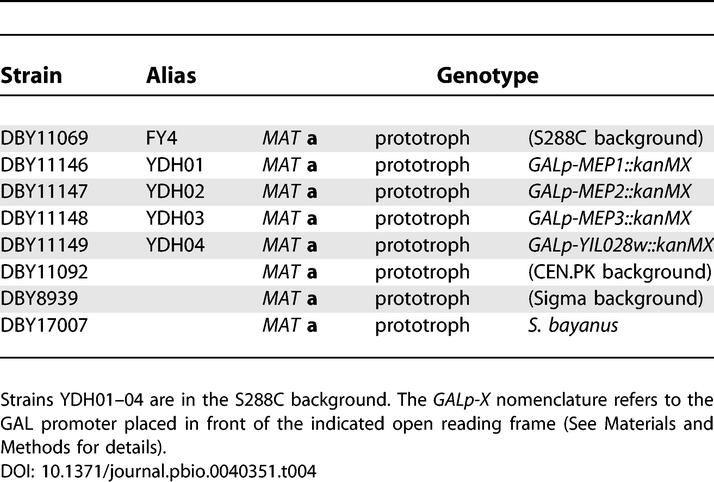
S. cerevisiae Strains Used in This Study

### Medium formulation.

Chemostats media used the phosphate-limited medium formulation from Saldanha et al. [5]. This base salt–mixed medium contained: 0.1-g/l calcium chloride dihydrate, 0.1-g/l sodium chloride, 0.5-g/l magnesium sulfate heptahydrate, 5-g/l ammonium sulfate, 0.98-g/l potassium chloride, 20-mg/l potassium phosphate, and 5-g/l glucose, plus vitamins and metals described previously [5]. Variations from this base medium are noted in the text and figure legends. The pH of the medium was 4.3.

### Chemostat.

The chemostat is a well-stirred fermenter vessel into which fresh medium is fed at a constant dilution rate, with culture effluent (containing also cells) removed at that same rate. Monod [9] demonstrated that metabolic homeostasis can be achieved in this system over a very broad range of dilution rates because microbes appropriately match their growth rate to the dilution rate set by the experimenter. When steady state is reached, all chemical fluxes through the chemostat must be balanced. This allows for precise experimental quantitation of nutrient utilization, growth yield, and waste-product excretion under well-defined physiological circumstances.

### Chemostat growth conditions.

An overnight culture grown in standard phosphate-limited chemostat media (see above) was used to inoculate a 300-ml working volume chemostat which was then grown in batch for 24 h at 30 °C (26 °C for S. bayanus). The chemostat was then run in continuous mode with a dilution rate of approximately 0.17 volumes per hour. After approximately 48 h, biomass measurements stabilized, indicating steady state. Chemostats were run for an additional 24 h to ensure steady state. At this point the chemostats were harvested and assayed for cell morphology, filtrate composition, nutrient consumption, and transcript abundance.

### Culture sampling.

Vacuum filtration was used to collect yeast cultures on filters that were subsequently quick frozen in liquid nitrogen. The filtrate was frozen at −20 °C for later analysis.

### Cell density and volume measurements.

Cell density and volume were measured daily using the Z2 automated cell counter (Beckman Coulter, Fullerton, California, United States). Chemostat culture was diluted into Isontone II buffer for counting. Daily measurements of cell density were taken using a Klett Colorimeter (Manostat, New York, New York. United States).

### Residual phosphate, residual glucose, and ethanol assays.

Residual phosphate was measured as previously described [46]. A calibration curve revealed the assay to be highly linear between 2 and 150 μM. Residual glucose and ethanol concentrations were assayed in the growth medium by enzyme-coupled NADH oxidation reactions (assay kits from R-Biopharm, Darmstadt, Germany). Assays were performed in triplicate for each filtrate.

### RNA isolation and labeling for microarrays.

Cultures were harvested for RNA by vacuum filtration onto nylon filters. RNA for microarray analysis was extracted from culture filtrate by the acid-phenol method, and cleaned using RNeasy mini columns (Qiagen, Valencia, California, United States). Cy3- or Cy5-labeled CTP (Perkin Elmer, Boston, Massachusetts, United States) was incorporated into amplified RNA using the Agilent Low RNA Input Fluorescent Linear Amplification Kit using half reaction volumes (Agilent, Palo Alto, California, United States). Labeled samples were analyzed for dye incorporation using a Nanodrop ND-1000 Spectrophotometer (Nanodrop Technologies, Wilmington, Delaware, United States).

### Microarray data acquisition and processing.

Each Cy3-labeled experimental cRNA sample was mixed with the Cy5-labeled reference cRNA and fragmented using the In situ Hybridization Kit (Agilent). The probes were hybridized for 17 h at 60 °C to an Agilent Yeast V2 Oligo Microarray. Microarrays were washed and then scanned with an Agilent G2505B Microarray Laser Scanner, and analyzed using Agilent Feature Extraction software. Resulting microarray intensity data were submitted to the PUMA Database (http://puma.princeton.edu) for archiving and analysis. These files can be accessed at the following URL: http://puma.princeton.edu/cgi-bin/publication/viewPublication.pl?pub_no=508. Additionally, the raw data files are available as Datasets S1–S9. Results were expressed as the log_2_ of the sample signal divided by the signal in the reference channel. Spots flagged as “outliers” during Agilent Feature Extraction were discarded.

### Mass spectrometry.

LC-MS/MS analyses were performed on a Finnigan TSQ Quantum Ultra triple quadrupole mass spectrometer (Thermo Electron Corporation, San Jose, California, United States), coupled with a LC-10A HPLC system (Shimadzu, Columbia, Maryland, United States). The instrument control and data analyses were performed through XCalibur software (version 1.4 SR1, Thermo Electron Corporation). The metabolites were detected in Selected Reaction Monitoring (SRM) mode allowing for scanning numerous metabolites in a single LC run. A complete list of the SRM parameters for every metabolite studied, as well as a full description of the LC-MS/MS method development and validation, has been given previously [47]. The MS instrumental parameters were: positive ionization mode, spray voltage 3,200 V, nitrogen as sheath gas at 30 psi and as auxiliary gas at 10 psi, argon as the collision gas at 1.5 mTorr, and capillary temperature 325 °C. Scan time for each SRM was 0.1 s with a scan width of 1 *m/z.*

Chromatography separation was achieved on a Luna aminopropyl (NH_2_) column (250 × 2 mm, particle size 5 μm; Phenomenex, Torrance, California, United States) at hydrophilic interaction chromatography (HILIC) mode, using a binary gradient solvent system. Solvent A is 20 mM ammonium acetate plus 20 mM ammonium hydroxide in 95:5 water:acetonitrile, [pH 9.40]. Solvent B is acetonitrile. The gradient was: t = 0, 85% B; t = 15 min, 0% B; t = 28 min, 0% B; t = 30 min, 85% B; t = 40 min, 85% B. Other LC parameters were: autosampler temperature 4 °C, column temperature 15 °C, injection volume 20 μl, and solvent flow rate 150 μl/min.

Just prior to LC-MS/MS analysis, the filtration samples were spiked with three isotopic-labeled internal standards at a final concentration of 1 μg/ml each. The internal standards were, L-alanine (U-13C3, 98%; 15N, 98%), L-glutamine (U-13C5, 98%; 15N2, 98%), and L-glutamate (U-13C5, 98%; 15N, 98%), all obtained from Cambridge Isotope Laboratories (Andover, Massachusetts, United States). Error reported in the figures was based on the internal standard that varied the most over a set of conditions. All other metabolite standards were obtained from Sigma-Aldrich (St Louis, Missouri, United States). The metabolite concentrations in the samples were derived by comparing the peak area of the corresponding chromatography trace with metabolite standards in a separate run (external calibration). Spiking also allowed direct quantitation of the relative amounts of amino acids inside cells and in the filtrates (i.e., excreted). Cell extracts were performed with a method adapted from previous methods for metabolite extraction [47,48]. Briefly, yeast cells harvested onto a filter were extracted twice with 100% methanol at −70 °C and then twice with 80% methanol at 4 °C. The isotope-labeled standards were added after the extraction process. In one experiment under nitrogen toxicity (1.3 mM potassium; 76 mM ammonium), we found in cell lysates the following amounts (nmoles per million cells): ala, 0.12; glu, 0.13; and gln, 0.03; for the filtrates: ala, 0.48; glu, 0.41; and gln, 0.31.

## Supporting Information

Dataset S1 The 13 mM K Array—Raw Data Raw data file produced with Agilent feature extraction software.
(6.9 MB XLS)

Dataset S2 The 10.6 mM K Array—Raw Data Raw data file produced with Agilent feature extraction software.
(6.9 MB XLS)

Dataset S3 The 8.0 mM K Array—Raw Data Raw data file produced with Agilent feature extraction software.
(6.9 MB XLS)

Dataset S4 The 5.3 mM K Array—Raw Data Raw data file produced with Agilent feature extraction software.
(6.9 MB XLS)

Dataset S5 The 2.7 mM K Array—Raw Data Raw data file produced with Agilent feature extraction software.
(6.9 MB XLS)

Dataset S6 The 2.0 mM K Array—Raw Data Raw data file produced with Agilent feature extraction software.
(6.9 MB XLS)

Dataset S7 The 1.3 mM K Array—Raw Data Raw data file produced with Agilent feature extraction software.
(6.9 MB XLS)

Dataset S8 The 0.65 mM K Array—Raw Data Raw data file produced with Agilent feature extraction software.
(6.9 MB XLS)

Dataset S9 Figure 2A Raw Array Data—Raw Data Raw data file produced with Agilent feature extraction software.
(410 KB XLS)

Figure S1 Cell Viability Does Not Decrease Significantly under Ammonium Toxicity Cells were grown in chemostat media with the indicated amount of potassium for 24 h in batch. After batch growth, approximately 300 cells were plated on YPD in triplicate. Colonies were counted after 2 d of growth at 30 °C, and the percent viable cells were calculated.
(61 KB PPT)

## Abbreviations

LC-MS/MS: liquid chromatography–tandem mass spectrometry
